# Ten simple rules for starting (and sustaining) an academic data science initiative

**DOI:** 10.1371/journal.pcbi.1008628

**Published:** 2021-02-18

**Authors:** Micaela S. Parker, Arlyn E. Burgess, Philip E. Bourne

**Affiliations:** 1 Academic Data Science Alliance, Seattle, Washington, United States of America; 2 School of Data Science, University of Virginia, Charlottesville, Virginia, United States of America; Carnegie Mellon University, UNITED STATES

## Introduction

Data science has emerged as a new paradigm for research. Readers of this journal might be tempted to say this is the research we have been doing all along. However, we contest that there is something fundamentally different in terms of the dimensions of data, diversity of disciplines, as well as the role of the private sector, than what has gone before. We take this position based on our collective experiences in, and observations of, calls to action around data science over the past 10 years. Those calls have resulted in many notable and successful responses from US universities.

### A working definition of data science

Defining data science is like defining the internet—ask 10 people and you get 10 different answers. What most would likely agree on, at a high level of abstraction, is that it draws from statistics, computer science, and applied mathematics to operate on data from one or more domains leading to outcomes not achieved otherwise. The extent to which domain knowledge is incorporated in the work of data science varies, but it is essential for achieving meaningful outcomes. Outcomes that have implications to us as humans and our collective communities and society that, in turn, need to be addressed as part of the data life cycle [1]. In short, data science transcends traditional disciplinary boundaries to discover new insights not owned by any one existing discipline, driven by endless streams of digital data with the promise of translation to societal benefit.

### Ten years of academic data science

The past decade has seen an explosion of data science centers, institutes, and programs appearing across the United States as universities increasingly recognize the importance and promise of data science to university research and education. It has been, and continues to be, an exciting time. But there are systemic challenges faced by these initiatives in the context of the higher education system. Some, but not all, of these challenges center around funding. Campuses fortunate enough to receive initial funding, often as a result of philanthropy or private sector investment, have some measure of sustainability, especially if these funds are in the form of an endowment. However, at most smaller colleges and universities, or those without a lucrative alumnus or local industry investor, just getting started with very limited funding can be daunting. And yet, every school is facing the reality that to truly prepare their student body for the expectations of 21st century employers, they must find a way to incorporate core critical thinking and data-intensive skills into nearly every discipline. This call to action challenges traditional disciplinary silos and begs for new models of higher education. The expectations of our future society will not leave this readership immune. How best to engage in this new paradigm?

In 2013, two foundations (the Gordon and Betty Moore Foundation and the Alfred P. Sloan Foundation) and three universities (the University of California, Berkeley, New York University, and the University of Washington) established a partnership to experiment with creating supportive environments for researchers using and developing data-intensive practices. Known as the Moore-Sloan Data Science Environments (MSDSEs), funding from the foundations helped to establish data science centers on each of the campuses: the Berkeley Institute for Data Science, the Center for Data Science at NYU, and the eScience Institute at UW. The partnership organized around working groups on cross-cutting topics viewed as critical to advancing data science in academia: career paths and alternative metrics, software development, education, reproducibility and open science, reflexive and reflective ethnography, and the role of physical space in collaboration. After 5 years, the partnership authored a lessons-learned paper “Creating Institutional Change in Data Science” [2] which includes the key elements that contributed to their successes and draws out some of their challenges along the way. This paper was followed by a formal evaluation of the MSDSE partnership [3], which includes a landscape survey of 17 other data science initiatives to find commonalities in approaches [4].

Since inception, the MSDSEs have been joined by countless other universities launching initiatives to grapple with the integration of data science in academia (examples in S1 Table). A handful of data science initiatives even pre-date or emerged at the same time as the three Moore-Sloan partners. These many initiatives, and many more not cited, continue to face a unique set of challenges due to their campus’ political, financial, and structural environments. Our consideration of their challenges and those we have faced directly ourselves lead us to recognize a set of global commonalities. We capture them here in the familiar Ten Simple Rules (TSR) format for simplicity, recognizing much more could be said.

We do so as representatives of these developments: MSP was formerly an Executive Director of the eScience Institute at the University of Washington and now works with data science centers nationwide through her leadership of the new Academic Data Science Alliance, AEB is Chief of Staff, and PEB is the Dean of the School of Data Science at the University of Virginia.

### Names and definitions

What do we mean by “academic data science initiative”? Data science is typically housed in a cross-departmental unit on academic campuses, such as an Institute, Center, or Program. More recently, Departments and Schools of data science are emerging when a degree is offered. While such formal academic units are necessary for many campuses, they risk not fully recognizing data science as cross-cutting, making it more difficult to move away from a sense of silos and hierarchies. The University of Virginia speaks to the kind of model for higher education called for by considering data science as a “School Without Walls,” an organization that has autonomy with other disciplines. Embracing this concept, we refer to all these efforts and organizations as “initiatives,” emphasizing that data science serves everyone and recognizing that everyone can contribute to the evolution of data-intensive research practices.

### Assumptions

We are assuming you, our readers, have already determined that a data science initiative would be a valuable addition to your campus, or you are indeed already part of a new initiative. Compelling examples of reasons to start a data science initiative have been highlighted elsewhere (e.g., [5]) and include:

- having an organizational unit to handle the emerging challenges of research software development and sustainability [6,7];
- incentivizing the use of, and providing training on, tools for reproducible and open research, data, and scholarship, e.g., Reprozip [8] and Git/GitHub, highlighted in this TSR: [9];
- establishing curricula for non-data science departments to integrate into courses [10];
- developing and running informal training programs for the researchers on campus who already feel left behind by the data revolution [11–13];
- creating a home for cross-disciplinary interest groups to work together on common challenges around data, e.g., [14], including scholarly discussion and actionable progress toward responsible data science, e.g., [15], and diversity, equity, and inclusion (DEI) in data science [16];
- and last but certainly not least, providing a space and culture for the data scientists, research software engineers, and consultants who will make this all happen [6,17].

Importantly, the reasons, and thus the approaches, must take into account your campus community’s needs and strengths, your university’s bureaucracy, and the political landscape of your campus. With this TSR, we offer what we believe are the most important considerations, some philosophical, some practical, as you develop and contribute to your initiative’s future.

## Ten simple rules

### Rule 1: Don’t try to own everything

Building a new data science initiative is all about partnerships and harnessing energy. Don’t try to take away the thunder from existing data science efforts on your campus. You will create undue competition and fail, or at the least dilute an institution-wide effort. Start by relationship building with the groups you are aware of and find areas where you could collaborate (recognizing that you may not even be aware of all the data science groups on your campus). Support existing work where added partners multiply impact and find the unmet needs to tackle as a leading organization, inviting others to join you. Show them what they have to gain: a positive and inclusive culture that draws people in [6] and serves students, researchers, and faculty with partnerships. Researchers faced with an avalanche of data from increasingly sophisticated instruments, models, and algorithms gravitate to data science. Where possible, direct them to other researchers for potential collaborations. In this way, your initiative becomes a network hub to and for researchers and a means to share best practices.

Partner with the information/information sciences, statistics, computer science, and other departments to offer courses or develop curricula, especially if you don’t want to start out with the heavy lift of organizing and offering a formal degree (assuming your institution would permit it). But be careful to not take away tuition revenue from existing programs by creating competing courses. While the negative effects of this will depend on the institutional funding model, it is likely where major resistance will lie. Rather, share in the burden and the rewards by partnering on curricula and teaching loads. Done right, your work will increase, not decrease, tuition revenue for everyone. Already there are examples of data science degree programs with up to four or more joint departments sharing the revenue and expenses. Recognize the strengths of these departments and partners, and build upon them.

Praise and elevate the work of people in your institution who are doing data science. Give them a platform and they will be your advocates; ignore their work and they will resent your efforts. Be the glue.

### Rule 2: Leverage champions to get buy-in from stakeholders

Have a faculty champion, a senior faculty person who has political sway and can bring in other faculty from diverse schools and colleges across the university. And someone who has the ear of the university leadership (e.g., Provost). Ideally, you have several champions who may form the basis for an executive or steering committee. Establish this committee and an external advisory board (EAB) at the beginning. The former brings ties to departments and schools across campus, the latter invests both intellectually and possibly financially. EAB members who are outside of academia can create bonds to other sectors, such as industry. Leverage their networks to engage with external communities (Rule #9).

Reach out to champions who have time and energy to invest in your initiative. They should be willing and able to do work, at least in the beginning. Often, associate deans are well connected to their peers and can contribute more than college deans or department chairs who have so many competing areas to manage. And, as noted with Rule #1, recognize those who put in the time and thought with you.

Use your champions to get as much buy-in from across the university as you can: reach out to the president, provost, deans, faculty, and administration. You are choosing to do something very different from anything they have worked on before—it’s more inclusive and more connected than anything on campus. Recognize that you will push the policies that they have worked very hard to write and interpret. Getting buy-in from the administration early means that they are also invested in your success and they know that you understand the complications in achieving your goals. Changing university policy is a slow, complex, political process—you will need all the allies you can get.

At the outset, or as interest fades, incentivize engagement from faculty with 1 or 2 months of summer salary or a spot on the executive/steering committee (with clear term limits). Consider negotiating a term of teaching release with their department to do X, where X could be heading up a new Special Interest Group, organizing a career fair or cross-campus data science summit, or developing new teaching tools around data science. This negotiation may be difficult unless you can demonstrate a win for the department from X (e.g., developing a data science curriculum for them). Show them what they have to gain and give them the wins when things succeed.

Maintaining goodwill with departments across campus will make it easier for your champions to promote your initiative in their departments. Have some pilot projects with these champions that can be pointed to when you make your case for formalizing your efforts into a campus-wide initiative. Early success stories will make the pitch for engagement from faculty easier. Then, popularize what you propose with the faculty senate and other governance bodies.

Finally, realize that champions are not only senior faculty. They are enthusiastic early-career faculty, data savvy staff, students, and postdocs across your campus. If you bring them in, they will be a huge win for your initiative. They are the bridges connecting everyone and tireless advocates. And they can help make a case for data science in departments that remain skeptical of the utility and impact of data-driven approaches in their fields or the value that a data science initiative adds to their own efforts in this space. Set clear and realistic expectations for their engagement. Postdocs, especially, are in a very time-limited and career-critical place. Be sensitive to their needs and don’t expect them to set aside their research and career demands for your initiative’s needs. As much as possible, find ways for them to contribute that also benefits their future career. Give them something meaningful they can put on their CV. Data science opens new career doors with substantial rewards. Your efforts will be somewhat of a *Catch-22* as you endeavor to keep them in a competitive market.

### Rule 3: Have a sustainability plan (and find funding)

Once you have secured champions and buy-in from stakeholders, but before you begin hiring the staff that you’ll need, have at least a rudimentary sustainability plan to share with them. You will more quickly attract expert staff when you demonstrate that you understand and are planning for sustainment, as well as a strong start. We will presume you have some initial balance of support from: tuition revenue, philanthropy, private or public sector investment, indirect cost return from grants, or if you are lucky, core budget lines. In short, build on these initial resources, demonstrate value, and sustainability will follow as it does in a high-demand marketplace.

When you first pitch for the establishment of your initiative, show the demand and back it up with budget projections. Don’t reinvent the wheel by doing all of your own research. Talk to leaders of data science initiatives on other campuses (e.g., S1 Table, the Academic Data Science Alliance can help connect you). There are over 450 data science degree or certificate programs in the US alone [18], and collaborative, cross-sector groups focusing on data are everywhere. Leverage the work they have done to show the benefit of a data science initiative, and how you will model your initiative to meet the demand. If your administration is still skeptical about data science as rigorous scholarship and its impact on research across campus, bring them examples of funding successes from similar types of institutions—peer pressure can be very effective! Once your initiative is up and running, the question will shift from “what will you do” to “what have you done.”

One approach to consider is providing consulting and training services to the campus. Data intensive practices touch nearly every discipline, but the degree to which students, faculty, and staff researchers are trained in these skill sets varies hugely across campus. Focus some of your efforts on serving these researchers with consultations, trainings, or as research partners. Faculty and staff, especially, don’t have the bandwidth to learn data science through formal curricula. Invite your staff to develop their teaching skills by becoming Carpentries instructors (https://carpentries.org/) or developing short, informal courses on common tools like Git/Github. These training opportunities demonstrate the value-add of your initiative to the greater campus community and your efforts may be rewarded with some core university funding. Partner with the Libraries (Rule #10) or other entities that are also creating these opportunities, but be careful that the role of your data science effort isn’t blurred with the mandate of university IT services or with a campus research computing organization.

The term “Research Software Engineer” (RSE) is sometimes used to describe professionals on campus who provide consultation services (though their primary role is in software development for research). RSEs are often housed in university IT, but many are also skilled researchers. Data scientists are distinct from RSEs, though there can be a lot of overlap in skill sets making the distinction difficult. Partner with your IT unit and RSEs to fill the cyberinfrastructure and software engineering needs of your projects (see Rule #10). As part of this partnership establish an understanding early on of the infrastructure needs, aside from people, that the IT unit and data science initiative will each provide. The IT department should be a collaborator and its staff your colleagues if data science is to reach its full potential on your campus.

Of course, there are other sources of funding that can be pursued in tandem and with partners: tuition revenue from new courses, fees from professional certificate programs, standard research grants to your staff, opportunities to partner with philanthropic organizations, and funding from the private sector (start an industry affiliates program).

Regardless of your sources of funding, from Day 1, track everything you do or support: every grant where your initiative is listed as a resource, where you’ve provided a letter of support, or where your core affiliates or staff are listed as PIs (Principal Investigators). Tally these dollars regularly. Track engagements with students, researchers across campus, and externally. Tally these “touches” to demonstrate the reach of your initiative and its impact on campus. Before asking for more university or college funding, collect kudos from everyone you’ve ever helped—a stack of anecdotes that say “we couldn’t have done this without you” from across campus has a big impact. And remember, all of this work can’t be done without support staff. Include them in the process and credit them as equal contributors to your efforts.

Budget for and contract with professional evaluators to help you track your successes and identify challenge areas. Do this sooner rather than later or you won’t have the baseline data to show progress.

### Rule 4: Hire a team, and support them

Data science has the power to change many, many people’s lives for the better, and for the worse (see Rule #7). It cannot be emphasized enough that data science initiatives prioritize hiring a diverse workforce who have the backgrounds and lived experiences to ensure the development and application of new technologies appropriately consider sensitive data and marginalized groups, and “do no harm.”

Don’t underestimate the staffing needs of a new initiative, both professional administrative staff and research staff. At minimum, plan to hire three professional administrative staff right at the beginning: a head of operations (Program Manager, or similar) who can grow into an Executive/Managing Director role and provide strategic advice in the years to come, a communications and/or events person, and a fiscal specialist. Consider joint hires, but recognize that a larger entity will always dominate the bandwidth of a shared staffer. For the head of operations and communications positions, select people who are intellectually curious about data science and want to make it accessible to all of your stakeholders. In particular, for the communications position, bring in someone with science writing experience who can write stories. Stakeholders—especially donors—need stories.

Equally important, target some initial funds to hire data science research and/or consulting staff. They can provide return on your investment both through consultation support to the university, demonstrating the value of your initiative to campus for future provostial funding requests, and/or through their own impactful research which can attract grant dollars. This means giving them PI status, or changing policies in departments and schools where this isn’t allowed. (Yes, really. The authors are baffled by departments who continue to stand in the way of grant dollars.) But have a clear plan for how these appointments will be sustained (Rule #3). Do they receive some initial funding but have responsibility for all or some of their own salaries over time? What are the expectations around fractional or full support?

Data-intensive research is by necessity a team sport. The team usually includes one or more members strong in the skill sets that define data science, in addition to subject matter experts. Rarely does one person, or even one “lab” group, have all the necessary skills needed to complete a successful data-intensive project. This is where your initiative comes in. Help domain researchers build the right team by match-making appropriate data scientists with the needed skill sets. Depending on the size of the project, this could start as a new joint hire. Importantly, recognize that the skills that define one data scientist are not necessarily the same as the skills that define another. A data scientist can be someone formally trained in statistics who picked up programming in their free time and data management from practical experience. They can be the social scientist who received some formal data science training (a minor, certificate, or advanced degree) and learned reproducibility tools from colleagues or informal training events. They can be the computer scientist who specializes in data visualization and has training in data ethics. The combinations are endless. Finding the right team members means not just advertising for a “data scientist”—it means knowing what you need and finding the right people (or, if you are only looking to expand your initiative, advertise more generally and build from who you find). Be careful not to look only in your own discipline—biologists can learn a lot about image analysis from astronomers, just as political scientists can learn and apply algorithms from genomicists [19].

As soon as you are able to grow the team, consider hiring an ethnographer. Typically, they are data scientists themselves doing the data science of data science, and they are invaluable for providing thoughtful, real-time feedback on your programs. Often they have their own consultations and original research to contribute (e.g., [20,21]).

Be transparent about your expectations and how the staff should contribute to the mission of your initiative. Recognize that different people will have different balances of job duties (development and maintenance of research, educational, and service projects) that work best for them. Manage and evaluate their work accordingly. Nurture passion projects and clearly articulate ties to your initiative’s strategic focus in public-facing documents and your internal groups (see Rule #8). This fuels collaboration internally as well as externally and will assist in your recruiting efforts when people know they will be valued and all aspects of their work will be recognized.

Hiring data science staff means you also need to think about their career paths. Promotion pathways and sustainability for data scientists and RSEs has been a topic of discussion for years (reviewed in [17]), yet there are disappointingly few examples of universities who have made real strides in this area until very recently [7]. Revisit the descriptions of current payroll titles and/or create new titles for data scientists that recognize and elevate the knowledge they have to contribute to campus. Similarly, career mentorship for staff data scientists is typically lacking, despite the need for more mentoring, not less, because these are relatively new positions [3,22]. We hope that with increasing successes for the universities who have chosen to value and fund careers for data scientists and RSEs, more universities will follow and increase both funding and recognition for these staff who enable so much of the research on campus.

### Rule 5: Recognize and elevate data, software, and workflow contributions

Hand in hand with career paths are the metrics by which all data-intensive researchers, faculty, and staff are evaluated for hiring, tenure, and promotion. Major pain points for faculty and staff working specifically in data science have to do with the current overemphasis on first-author journal publications. Data and software intensive research can result in months or years of data curation, software design, and data management/analysis workflow developments that are not easily published in traditional journals. And much of the work of a data scientist is “invisible” [23], especially within a new and growing organization. “Invisible” work includes maintenance of software (which is often underbudgeted, if budgeted for at all) and training or consultations for a seemingly endless flow of people who drop in, which do not count as much toward academic advancement as grants and papers. Ironic since without collaboration the research would not happen, so why not measure and reward it? If professional staff advancement is the goal, be sure to include all of the invisible work as part of the evaluation process.

Joint faculty hires across departments are already challenging because different fields can have different expectations and measures of success: A few first author papers in high-impact journals is expected in some departments, whereas others are looking for many submissions to conference proceedings, and some place more or less value on single authorship. The above pain points are amplified for joint hires in data science, where trying to explain to a domain committee member the importance of data science work (and vice versa) makes attaining tenure or promotion incredibly difficult.

Policies and metrics of success in higher education need to change so that open science, software, and data citations become recognized as equal partners to publications on CVs, and team members are recognized for their contributions, not their position on the author list [7]. Your champions can help by steering the metrics on hiring/tenure/promotion committees, but changes will happen faster if these ideas are echoed from above. It is increasingly important for university and college leadership to recognize that the metrics of success for research in academia are changing, and not just around data science. It’s time for university policies around hiring/tenure/promotion to reflect how research and discovery get done. Data science can lead the way.

And finally, colleges and universities must signal their support by prioritizing research software in core budgets. Development and maintenance of software that drives discovery is critical and essential in our current research landscape. It is unreasonable and unsustainable to expect individual PIs to earmark grant funds for software maintenance (if it is even allowed by the granting agency) when this software supports and drives discoveries across campus. Institutional funds for campus-wide resources, such as expensive journal subscriptions and the infrastructure needed to support and promote them, ought to be redistributed to include basic software maintenance, in collaboration with the Libraries and IT (Rule #10).

### Rule 6: Focus on interdisciplinarity, but don’t overdilute

Interdisciplinarity is the essence of data science. Be sincere about your interdisciplinary efforts, but recognize when your core researchers have strength in a particular area and double down on it. Trying to help everyone in every field across your campus at once isn’t realistic or tenable. Start with the expertise of your staff and affiliates and build from there. It’s okay to be known for a subset of disciplines where data science is applied, for example, in Earth science, biomedicine, or sociology. Focusing on your research strengths will help attract grant money. And as a university, you are ultimately part of a supply chain feeding data savvy researchers and thought leaders into society. You will never be able to meet all of the demand in every discipline, so figure out what to supply based on the strengths of your staff and institutional collaborations. As your successes grow, hire in the areas where you have gaps in expertise or domain knowledge and grow into new collaborations over time.

One avenue to broaden your community of collaborators and thus your interdisciplinarity is a postdoc fellowship program. Setting aside funds for postdocs to propose projects that cross disciplines will be rewarded by increased visibility of your initiative and increased collaborations, creating more champions (Rule #2). But be aware that data-savvy postdocs will typically command a higher salary than their peers (ignoring for a moment that all postdocs are underpaid). Budget for these accordingly, and be prepared to negotiate with departments, especially in disciplines without large budgets who try to ensure equity across their postdoc population. In the more lucrative fields (e.g., computer science), you may need to arrange for an additional kick-in from the department to bring the salaries up to the level of their peers. Match a domain and a methods mentor for the postdoc project (or better, have the postdoc identify and engage the faculty mentors themselves). This kind of relationship building creates bridges between departments, with your initiative at the nexus. Faculty in departments across your campus will see the value of engaging with your initiative. And some may learn to appreciate data-driven approaches if they have been reticent to bring data science into their work in the past.

### Rule 7: Emphasize responsible data science

If interdisciplinarity is the essence, responsibility should be the pillar of data science. Responsible data science isn’t just about providing an ethics course or discussion group. Ethical thinking and societal perspectives should be infused in your culture and every project you work on. It is a way of thinking that covers all data science research from conceiving of a project to the dissemination of the final product. This will become increasingly important as data science further captures the imagination of all stakeholders, while at the same time high-profile nefarious activities continue to threaten the integrity of the field. The potential for biases to propagate in algorithms and artificial intelligence (AI), such as facial recognition with its intrusive and racially biased outcomes and the recent surge in “bad” data and public misinformation due to the Coronavirus Disease 2019 (COVID-19) pandemic, highlights the need to provide much more rigorous training in ethics and social contexts throughout the data life cycle [1]. While this kind of training and context is often referred to as data science for the public good, it should be front and center in all data science projects and initiatives that have the potential to use data from, or impact the lives of, individuals or groups. These goals can only be achieved if the team reflects the diversity of the communities their work will impact, and with partnerships and all stakeholders at the table, bringing together STEM and humanities such that a virtuous cycle of human impact is fed back into how data are collected and analyzed. And while data science moves quickly, projects must “move at the speed of trust” to carefully apply techniques and incorporate feedback and input from diverse groups (https://www.blackspace.org/manifesto).

As you think about the programs your initiative can offer campus, use the knowledge base of your research staff, postdocs, and students to focus on opportunities that play to their strengths and allow your projects to model responsibility. Bring people together around a tool, challenge, event, or idea that emphasizes these values and demonstrates practical applications of responsible and ethical approaches to data science. Some examples: XDs such as ImageXD (https://bids.berkeley.edu/research/image-xd) and TextXD (https://bids.berkeley.edu/research/textxd), Hackweeks [11] (https://uwescience.github.io/HackWeek-Toolkit/), Datapaloozas (e.g., Health Datapalooza https://academyhealth.org/events/2020-02/2020-health-datapalooza, MassCUE Datapalooza https://www.masscue.org/event/masscue-datapalooza-2020/), and Women in Data Science events (https://www.widsconference.org/).

### Rule 8: Establish a set of guiding principles

As a new initiative, you will be tempted to say “yes” to every request. You will quickly get a sense of the needs of your campus and rapidly expand engagement and goodwill. But early on, be sure to establish your MVV (mission, vision, values) and revisit these annually. From your MVV, develop a set of guiding principles that can create boundaries and help you recognize when a project or request is out of scope. A few examples include: transparency (encourage data and software projects to be open source), reproducibility (support and train for reproducible workflows for all projects), emphasize projects that are for the public good (Rule #7), or support the idea of data science for all by steering resources toward domains outside of computer science and statistics. While every project may not address every one of your guiding principles, bringing everything that you do back to those guiding principles will help you shape a voice for your initiative that all your staff and affiliates understand, support, and echo across the campus. Knowing your focus will also help you build in time, by managing load and job expectations, and provide guidance to allow your people to develop and nurture new data science ideas and programs. And down the road, it will help you and everyone on your team to say “no” to requests that fall too far outside these principles. With finite resources, your growing reputation will mean the inability to meet the demand (see Rule #6). Be selective in what you do to match your MVV and partner with other groups on campus to direct requests elsewhere.

### Rule 9: Engage with external communities

Data, and thus data-intensive research, is pervasive. It drives business, shapes policy-making, and influences how we see each other and ourselves in society. Not surprisingly, the private sector, government, and nongovernmental organizations (NGOs) are all looking for talent and partnerships. Engage with them. They have a lot to offer and are often ahead of where we are in academia. But recognize that all external communities move at different paces and have different agendas; align yourself appropriately. Done correctly, these partnerships can be a win-win, bringing in funding and new data sets while supporting the development of new technologies and launching the careers of your students and postdocs. From the partner’s perspective, they gain access to a talent pipeline, a host of research expertise, and the ability to align with projects in keeping with a university’s mission, notably projects for the public good. One great example is the Data Science for Social Good program, pioneered at the University of Chicago (http://www.datasciencepublicpolicy.org/) and then picked up (with modifications) at Georgia Tech (https://ptc.gatech.edu/dssg), Carnegie Mellon (https://www.dssgfellowship.org), and the eScience Institute at the University of Washington (https://escience.washington.edu/dssg/).

### Rule 10: Leverage core service groups

Libraries have been in the information business for centuries, long before computing and long long before the data revolution. Leverage their expertise and the physical spaces they occupy (e.g., [24]). Consider an easy lift: jointly funding some data services support staff. Or be bold and work together to use the library spaces as a one-stop shop for all data and information needs. Many libraries are converting to collaboration spaces as physical resources (such as print media) are moved online or offsite, and library administrators seek creative uses of their physical space. Repurposed library spaces are a great option for a data science initiative: Libraries are considered “politically neutral” and are habitual places where students and researchers seek information and help. Having a neutral location can be critical to getting buy-in from multiple departments and your initiative will be seen less as a territory-grab by one or more existing departments. Location, location, location.

Similarly, before campus IT groups were tasked with managing email servers, they spent a substantial amount of staff time providing consultations and supporting research compute needs. Now, as email management moves to decentralized services like Gmail, many IT groups have diversified (enterprise IT, research IT, educational IT). While research computing isn’t always under a campus IT group, nearly every school now provides some amount of research support again, with on-premises high-performance computing (HPC) consultations or connecting and enabling the use of Cloud services (e.g., https://cloudmaven.github.io/documentation/index.html). Reach out to them early to involve them in the collaborative process and do what you can do together. Excellent examples of such partnerships include: DS3 at NYU (Data Science and Software Services; https://cds.nyu.edu/ds3/) and Northwestern IT Research Computing Services (http://www.it.northwestern.edu/research). Here again, depending on the structure of the IT department and their mandate, partnerships may need nurturing. Relationship build and develop a common purpose. Offering to support shared staff hires (e.g., cloud computing support) can get you a seat at the table with access to collaborators and pilot programs. Some IT groups may be in a position to support maintenance of more mature software (with additional budget from the university). Consider developing a pipeline for software developments within your initiative that can be supported by IT, with university funding.

Data science initiatives in higher education, together with the Libraries and IT, can serve to match-make research partners and provide training to accelerate impactful research across campus. Perhaps most importantly, data science initiatives can promote responsible data science projects and products so that data-intensive researchers are recognized for their critical roles in establishing just outcomes from the greater research community.

## Conclusion

The role that data and data science is playing and will continue to play in society is without question. This readership, while being savvy when it comes to data, should not ignore the influence that data science will have on the future of our fields. These rules are intended to help you engage in this maelstrom. Some in the field of biology may argue that data science is just a new word for bioinformatics. However, the emergence of data science is very different from bioinformatics in its scope—touching nearly every discipline and reaching nearly every organization across sectors from industry to government to academia. What is also clear is that data science represents a higher degree of interdisciplinarity and opportunity for collaboration than anything that has gone before. Institutions of higher education are coming to realize both the potential and the challenges that data science brings to their campuses. Starting and sustaining a data science initiative is not an easy task, but the reward is in the path that leads to coordinated and deeper integration of responsible and thoughtful data-intensive practices across campus. The authors acknowledge that there are many paths to success, some we inevitably didn’t cover. We hope these rules offer a starting point and some guidance for our readers to learn more.

## Supporting information

S1 Table Examples of data science initiatives launched over the past 10 years. (DOCX)
