# Supplementary material for: Ten simple rules for starting (and sustaining) an academic data science initiative
Source: PLoS Comput Biol. 2021 Feb 18;17(2):e1008628. doi: 10.1371/journal.pcbi.1008628 (PMC7891724; doi:10.1371/journal.pcbi.1008628)
Supplement: S1 Table — (DOCX) [file pcbi.1008628.s001.docx]

**S1 Table. Examples of data science initiatives launched over the past 10 years**

| **Organization Name** | **Inception** | **Campus** |
| --- | --- | --- |
| The Computation Institute | 2000* | University of Chicago |
| eScience Institute** | 2008 | University of Washington |
| *The Fourth Paradigm: Data-Intensive Scientific Discovery, published 2009* | | |
| Institute for Data Intensive Engineering and Science | 2012 | Johns Hopkins University |
| Data Science Institute | 2012 | Columbia University |
| Berkeley Institute for Data Science** | 2013 | University of California, Berkeley |
| NYU Center for Data Science** | 2013 | New York University |
| Information Initiative | 2013 | Duke University |
| Data Science Institute | 2013 | University of Virginia |
| Goergen Institute for Data Science | 2013 | University of Rochester |
| Stanford Data Science Initiative | 2014 | Stanford University |
| Michigan Institute for Data Science | 2015 | University of Michigan |
| Institute for Data, Systems, and Society | 2015 | Massachusetts Institute of Technology |
| Data Science Initiative | 2015 | Northwestern University |
| Translational Data Science Institute | 2015 | The Ohio State University |
| Harvard Data Science Initiative | 2017 | Harvard University |
| Center for Data and Applied Computing | 2018 | University of Chicago |
| Northwestern Mutual Data Science Institute | 2018 | Marquette University and the University of Wisconsin-Milwaukee |

School and Departments are not included in this list. *Replaced by the new Center in 2018. **Partners in the Moore-Sloan Data Science Environments
